# Five-year prospective multi-center cohort study of patients with giant cell arteritis in Greece

**DOI:** 10.31138/mjr.29.2.103

**Published:** 2018-06-29

**Authors:** Christina Tsalapaki, Eleni Nikitopoulou, Kyriaki A. Boki, Dimitrios Boumpas, Petros P. Sfikakis, Georgios Vosvotekas, Paraskevi V. Voulgari, Dimitrios Vassilopoulos

**Affiliations:** 1 Joint Rheumatology Program, National and Kapodistrian University of Athens, School of Medicine, Athens, Greece,; 2 Clinical Immunology-Rheumatology Unit, 2^nd^ Department of Medicine and Laboratory, Hippokration General Hospital, Athens, Greece,; 3 Rheumatology Unit, Sismanoglio Hospital, Athens, Greece,; 4 4^th^ Department of Medicine, Attikon Hospital, National and Kapodistrian University of Athens, School of Medicine, Athens, Greece,; 5 First Department of Propedeutic Internal Medicine, National and Kapodistrian University of Athens School of Medicine, Athens, Greece,; 6 Private practice, Thessaloniki, Greece,; 7 Rheumatology Clinic, University of Ioannina, Ioannina, Greece

**Keywords:** Giant cell arteritis, glucocorticoids, relapses, comorbidities, methotrexate, osteoporosis

## Abstract

Giant cell arteritis (GCA) is the most common systemic vasculitis in the aged population associated with significant morbidity due to the long term administration of corticosteroids and the presence of various comorbidities. Data regarding its current treatment modalities, comorbidities, morbidity and mortality in Greece are limited. In this multi-center, prospective study that begun at the end of 2015 patients with newly diagnosed GCA according to the modified 1990 ACR criteria, as well as individuals with established or relapsing disease have been included. During the 1^st^ phase of the study that is still ongoing, data are being collected concerning demographic and clinical characteristics of the patients, treatment at the onset of the disease and at relapses, relapses, adverse events of therapy, comorbidities, hospitalizations and deaths. During the 2^nd^ and 3^rd^ phase of the study patients will be reevaluated 2 and 5 years after their 1st evaluation. The study is expected to provide valuable data regarding the current clinical characteristics, comorbidities, therapeutic regimens used, relapse rate, morbidity and mortality of patients with GCA.

## INTRODUCTION

Giant cell arteritis (GCA) is the most common type of systemic vasculitis,^1^ typically affecting individuals older than 50 years, with an estimated incidence ranging between 0.1 to 33 cases per 100,000.^2^ Data regarding its prevalence and characteristics in the Greek population are limited. The latest available data indicate a prevalence of 0.08%^3^ while there are no long-term data available regarding its course, current treatment modalities, comorbidities and mortality.

GCA is a heterogeneous disease often not conforming to a single clinical presentation. Ischemic tissue damage, which is the most serious complication, results in vision loss in 15–20% of cases and requires urgent treatment.^1^ Glucocorticoids remain the mainstay of treatment for GCA and should be initiated promptly. Improvement of headaches, malaise, fever and polymyalgia rheumatic symptoms are often dramatic. While glucocorticoids are highly effectively at controlling systemic inflammation and preventing acute damage (i.e. vision loss), they fail to cure the disease or to induce long-term, treatment-free remission.

Data from epidemiological and retrospective studies indicate that 40–60% of GCA patients will relapse during follow-up, therefore requiring prolonged treatment courses.^4,5^ Approximately 50% of the relapses will take place during the 1^st^ year of follow-up, while 36% of patients will flare more than 2 times.^5^ Most frequent symptoms at relapse are polymyalgia rheumatica, headaches, scalp tenderness, jaw claudication and vision disturbances.^5^ Due to the frequent relapses, approximately 40% of patients do not manage to discontinue corticosteroids,^4^ resulting in long-term exposure and increased risk of adverse events. These include osteoporotic fractures, cataracts, diabetes and vulnerability to infections in an aged population.^6^ Data from a large multi-centric study showed that the long-term use (>12 months) of high doses of corticosteroids (> 10 mg/day) was associated with an increased risk of deaths due to serious infections.^7^ Despite these findings, a number of unanswered questions regarding the current state of treatment, the rate of side effects, the rate of comorbidities and the long-term survival of these patients remain unanswered.

## AIM OF THE STYDY

The aim of this multicenter, prospective cohort study is to evaluate the clinical characteristics, course, treatment efficacy, comorbidities, morbidity and mortality in Greek patients with GCA.

## METHODS

Since the end of 2015, a prospective epidemiological study supported by the Greek Rheumatology Society has begun, and so far, 84 patients have been enrolled. Participating centers include academic and non-academic rheumatology clinics, National Health System out-patient clinics and private offices. Ethical approval has been obtained by the local institutional boards of participating centers.

Patients newly diagnosed with GCA according to the modified 1990 American College of Rheumatology (ACR) classification criteria as well as patients with established or relapsing GCA have been included in the study.

The design of this prospective study includes 3 successive phases.

During phase 1 of the study, which is still ongoing, the following data are being collected:

- demographic and clinical patient characteristics
- current or previous treatment(s)/dose (including corticosteroids, non-biologic and biologic immunosuppressives)
- adverse treatment events
- comorbidities
- relapses (relapse is defined as new disease activity after a period of remission, or worsening disease activity that occurred during follow up)
- hospitalizations and
- deaths

Data are entered either in a printed form, or electronically via a specially designed portal (rheumstudygrps. gr), where participants log in by using personal codes provided to them by website administrators. Patients are registered by their first name/surname initials and at the end of each registration, a unique verification code is provided. Only the study coordinator has full access to data entered by all participants. Each participant had access to the data entered by his/her own center and could edit them until the closing date of this phase.

During the second phase of the study, all patients from the initial cohort will be re-evaluated 24 months (2 years) after their 1st evaluation. Data collection will be performed by the same methods that were used in phase 1 (printed and web-based form). In this phase, patients are registered exclusively with the unique verification code provided during phase 1.

During this third phase, a 3rd evaluation of the same cohort (5 years after the 1^st^ and 3 years after the 2nd evaluation) will be performed. Data collection will be done with the same methods that were used in phase 2 (printed and web-based form).

### Statistical analysis

All analyses will be performed with the use of Microsoft Excel 2013 and IBM SPSS Statistics v.20 software. Data will be analyzed by descriptive statistics. Demographic and descriptive continuous variables will be expressed as mean (standard deviation, SD) and median values (interquartile range, IQR). Categorical variables will be expressed as percentages. Chi square or Fisher’s exact test will be used for comparison of dichotomous and Mann-Whitney or t-test for continuous variables. Binary logistic regression analysis will be performed in order to identify variables associated with relapses, serious infections, hospitalizations, corticosteroid discontinuation and death.

## ANTICIPATED BENEFITS

This is the first multi-center prospective study of such scale in Greece that is anticipated to provide valuable data regarding the clinical characteristics, co-morbidities, therapeutic regimens used (including their efficacy, safety, rate of discontinuation), relapse rate, morbidity (hospitalizations, infections, malignancies) and mortality of patients with GCA.
